# Digital PCR for Detecting Loss of *BAP1* and *MTAP* in Pleural Fluids of Mesothelioma Patients: Perspectives in the Diagnosis

**DOI:** 10.1111/pin.70172

**Published:** 2026-09-27

**Authors:** Giulia Rosini, Greta Alì, Rossella Bruno, Monica Cipollini, Rudy Foddis, Alessandra Celi, Roberto Silvestri, Filomena Rea, Vittorio Aprile, Marco Lucchi, Federica Gemignani, Stefano Landi

**Affiliations:** ^1^ Genetics‐Department of Biology University of Pisa Pisa Italy; ^2^ CNR Institute of Neuroscience Pisa Italy; ^3^ Unit of Pathological Anatomy University of Pisa Pisa Italy; ^4^ Department of Translational Research and of New Technologies in Medicine and Surgery University of Pisa Pisa Italy; ^5^ Cardiac, Thoracic, and Vascular Department, Division of Thoracic Surgery University Hospital of Pisa Pisa Italy; ^6^ Department of Surgical, Medical and Molecular Pathology and Critical Care Medicine University of Pisa Pisa Italy

**Keywords:** BAP1, digital PCR, gene copy number, immunohistochemistry, liquid biopsy, MTAP, pleural fluids, pleural mesothelioma

## Abstract

Pleural mesothelioma (PM) often presents loss of *BAP1* and *MTAP* genes. Digital PCR (dPCR) can accurately measure gene copy numbers (CNs). We hypothesized that cell‐free DNA (cfDNA) extracted from the supernatant of pleural fluids (SPFs) could allow an efficient detection of these genetic events. *BAP1/MTAP* CN was measured with dPCR in SPFs and in solid tumor biopsies (STBs) of 37 PM patients. Results were compared with immunohistochemistry (IHC) carried out in STBs. IHC alone detected BAP1/MTAP‐losses in 32/37 (86.5%). However, the sensitivity improved to 34/37 (91.9%) when low CN of *BAP1/MTAP* was included. Among 20 patients presenting SPFs, dPCR alone detected a low CN in 18 samples (sensitivity = 90%). Finally, this two‐gene signature was applied on SPFs in a prospective series of six consecutive patients presenting an undefined diagnosis (negative for PM) and two showed reduced CN. After 7 months of follow‐up, these subjects were further re‐evaluated for the persistence/worsening of the symptoms and were diagnosed for PM. We highlighted dPCR's potential for detecting subtle *BAP1/MTAP* losses in SPFs of PM patients. This appears as a complementary approach for a more accurate diagnosis in specific contexts, when high‐risk patients receive negative results after IHC analysis.

## Graphical abstract

*BAP1/MTAP* gene losses were measured with dPCR in solid tumor biopsies (STBs) and in supernatant of pleural fluids (SPFs) of mesothelioma patients. Results were compared with immunohistochemistry (IHC) carried out in STBs. A good concordance between the methods was found and, interestingly, their combination seems to ameliorate the identification of PM patients. the CNV's analysis in SPFs could be a complementary approach for a more accurate diagnosis in specific contexts, when high‐risk patients receive negative results after IHC analysis.

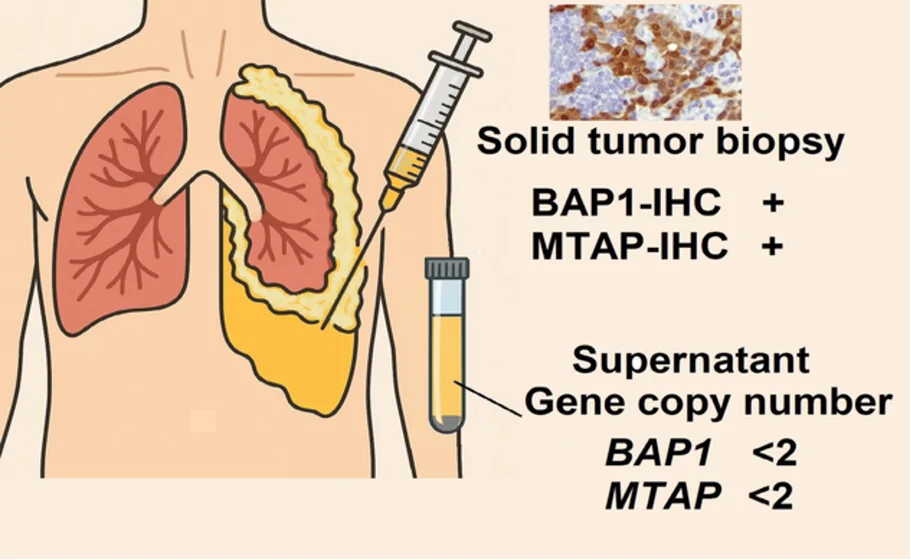

## 1 Introduction

Pleural mesothelioma (PM) is a rare and aggressive tumor originating from the mesothelial cells of the pleura. The primary etiological factor is asbestos exposure, although other contributors, such as prior chest irradiation and genetic predisposition, have also been identified [1]. Histologically, PM is classified into three main subtypes: epithelioid, biphasic, and sarcomatoid, with the epithelioid subtype being the most common variant and generally associated with longer overall survival [2]. Despite significant advances in surgical techniques, chemotherapy, and immunotherapy, the prognosis of PM remains poor because of delayed diagnosis and limited therapeutic efficacy [3, 4].

A large proportion of PM specimens exhibit loss of nuclear BRCA1‐associated protein 1 (BAP1) expression, reported in up to 77% of cases [5], and/or loss of the tumor suppressor proteins p16^INK4A^ and p14^ARF^ (both encoded by alternative transcripts of the *CDKN2A* gene), reported in up to 80% of cases [6]. These alterations result primarily from homozygous deletions involving the corresponding chromosomal regions, 3p21.1 and 9p21, respectively, or from hemizygous deletions accompanied by additional genetic or epigenetic events that inactivate the remaining allele [7, 8].

BAP1 exerts tumor‐suppressive effects through the regulation of several cellular pathways. In the nucleus, it influences gene transcription, chromatin remodeling, cell‐cycle progression, differentiation, and metabolism, while also promoting DNA repair by facilitating homologous recombination [9, 10, 11, 12]. In the cytosol, BAP1 appears to participate in calcium‐mediated cell death, characterized by increased mitochondrial Ca^2+^ levels, subsequent release of cytochrome c into the cytosol, and activation of apoptosis [13]. Somatic BAP1 inactivation is relatively frequent in PM and less prevalent in most other malignancies. Clear‐cell renal carcinoma shows the second‐highest prevalence, at approximately 15% [14]. BAP1 is typically assessed by immunohistochemistry (IHC), with nuclear staining indicating detectable expression of the protein. Loss of nuclear BAP1 staining is a reliable, rapid, and cost‐effective surrogate for biallelic BAP1 inactivation. However, retained nuclear staining does not necessarily indicate an entirely wild‐type *BAP1* locus, because some pathogenic alterations may not result in loss of detectable protein expression [15, 16].

Another important genetic event, although less specific to PM, is the inactivation of p16^INK4A^ and p14^ARF^, two tumor suppressor proteins involved in cell‐cycle control, particularly at the G1/S checkpoint. The *CDKN2A* locus is frequently deleted in PM, but *CDKN2A* deletion also occurs in many other cancers, including approximately 58% of glioblastomas, 40% of esophageal carcinomas, 32% of melanomas, and 28% of pancreatic adenocarcinomas [17, 18, 19].

The clinical manifestations of PM, including chest pain, dyspnea, and pleural effusion (PE), are nonspecific and may mimic other respiratory conditions, thereby complicating early detection [4]. Standard imaging techniques are often insufficient to establish a definitive diagnosis, making solid tumor biopsy (STB), usually obtained by video‐assisted thoracoscopy, essential for reliable diagnosis. However, this procedure is invasive and unsuitable for repeated monitoring of disease progression and therapeutic response [20]. Because more than 70% of PM cases present with PE that may contain tumor cells, pleural fluid (PF) analysis has been proposed as a less invasive and potentially more cost‐effective adjunct to tissue‐based diagnosis [6, 21].

The use of cytological analysis for the diagnosis of PM has historically been controversial. Early studies reported a sensitivity of approximately 30% [22], whereas more recent studies have reported sensitivities of up to 70%, following improvements in cytopathological expertise and the use of ancillary techniques [23]. The British Thoracic Society guidelines recognize cytological examination with IHC as an adjunct to, rather than a replacement for, histological diagnosis stressing the importance of STBs in providing a definitive diagnosis for PM [24]. While IHC analysis is based on classical markers such as calretinin, WT1, podoplanin, cytokeratin 5/6, GATA3, and vimentin, subsequent studies have shown that loss of BAP1 expression and/or homozygous deletion of *CDKN2A* in cytological or tissue specimens can achieve very high specificity for distinguishing benign from malignant mesothelial proliferations, thereby supporting a definitive diagnosis of PM [25, 26, 27, 28]. Homozygous deletion of *CDKN2A* is generally detected by fluorescent in situ hybridization (FISH). However, this method can be technically demanding and may yield false‐negative or false‐positive results if it is not appropriately performed and interpreted [29, 30]. Loss of methylthioadenosine phosphorylase (MTAP) protein expression, assessed by IHC, has therefore been proposed as a practical surrogate for homozygous deletion of the *CDKN2A* locus [31]. Indeed, 9p21 deletions frequently involve the *MTAP* gene, which is located adjacent to *CDKN2A* [32, 33, 34].

The loss of BAP1 and MTAP protein expression resulting from deletion of their corresponding chromosomal loci might also be investigated by measuring gene copy number (CN). We therefore hypothesized that digital PCR (dPCR)‐based measurement of *BAP1* and *MTAP* CNs in PF samples could represent an initial step toward the use of PF molecular analysis as an adjunct to conventional diagnostic procedures. dPCR has shown good analytical performance in detecting reduced CNs of *BAP1*, *CDKN2A*, and *MTAP* genes in formalin‐fixed, paraffin‐embedded PM tissues, including in studies that compared its results with those obtained using the reference methods of FISH and IHC [35, 36]. dPCR has also been successfully applied to the detection of *HER2* amplification in breast cancer [37]. Of particular interest, dPCR enables the analysis of cell‐free DNA (cfDNA) extracted from the supernatant of pleural fluid samples (SPFs), thereby expanding the range of minimally invasive approaches that may support the diagnostic work‐up. The clinical significance and predictive value of a reduced CN in SPF of PM patients is, at the present time, unknown and it must be established in appropriately designed studies.

In order to provide initial answers to these questions, the goal of the present study was to determine the prevalence of low *BAP1* and *MTAP* CNs in STBs and in cfDNA extracted from SPFs of PM patients. dPCR results obtained in STBs were also compared with the corresponding BAP1 and MTAP immunohistochemical findings. To this end, we used dPCR to assess *BAP1* and *MTAP* CNs in STBs from 37 patients with confirmed diagnosis of PM and, when available, in the corresponding SPFs from 28 of them. We then explored the performance of different testing strategies, based on IHC alone, dPCR alone, or an integrated analysis of the two methods, in detecting BAP1‐ and MTAP‐related alterations in STBs and SPFs. Moreover, we evaluated *BAP1* and *MTAP* CNs in SPFs from six individuals undergoing clinical follow‐up who were considered at increased risk of PM because of previous asbestos exposure. These individuals presented with PE at the time of thoracoscopy, but PM was initially excluded.

## 2 Materials and Methods

### 2.1 Patient Recruitment and Sample Collection

All patients underwent thoracoscopy for diagnostic purposes at the Unit of Thoracic Surgery of the University Hospital of Pisa between February 2021 and June 2023 and were subsequently diagnosed with PM, or phlogosis. The diagnosis of PM was established by the pathological anatomy laboratory of the same hospital, hereafter referred to as “laboratory P.” As part of routine clinical practice, laboratory P established the diagnoses using standard procedures in accordance with the ERS/ESTS/EACTS/ESTRO clinical guidelines, including IHC analysis for calretinin, WT1, podoplanin, cytokeratin 5/6, GATA3, and vimentin. In addition, laboratory P performed IHC analysis of BAP1 and MTAP and FISH analysis of *CDKN2A* in STBs and in the cell pellet from PFs. This cohort consisted of 37 patients aged 60–87 years, as summarized in Table 1. Patients with phlogosis consisted of six individuals all male aged 70–83 years.

**Table 1:** Main information of PM patients.

Sample ID	Histotype	Age	Gender	Stage	Exposure
PM_01	B	79	M	IV	U
PM_02	E	77	F	III	U
PM_03	B	60	M	III	+
PM_04	E	80	M	I	+
PM_05	E	70	M	III	+
PM_06	E	84	M	II	L
PM_07	B	76	M	IV	+
PM_08	B	87	F	II	−
PM_09	E	65	M	II	+
PM_10	E	83	M	II	+
PM_11	E	84	M	I	U
PM_12	E	70	M	II	L
PM_13	B	75	M	I	U
PM_14	B	71	M	N/A	+
PM_15	E	74	M	I	+
PM_16	B	79	M	I	U
PM_17	B	69	M	I	+
PM_18	E	71	M	III	U
PM_19	E	78	M	I	L
PM_20	E	69	M	I	+
PM_21	E	80	M	IV	U
PM_22	E	78	M	II	U
PM_23	E	84	M	II	U
PM_24	E	74	F	II	L
PM_25	E	86	M	II	U
PM_26	E	80	M	N/A	U
PM_27	E	77	F	N/A	−
PM_28	E	74	M	IV	L
PM_29	E	75	M	II	+
PM_30	B (mostly S)	72	M	II	L
PM_31	E	82	M	II	+
PM_32	S	75	M	III	+
PM_33	E	79	M	I	L
PM_34	B (E 85%–S 15%)	80	M	I	+
PM_35	E	60	M	N/A	U
PM_36	E	78	F	N/A	L
PM_37	E	70	M	N/A	U

*Note:* For histotypes: B = biphasic; E = epithelioid; S = sarcomatoid. For the exposure to asbestos: U = uncertain; +: exposed; −: not exposed; L: likely exposed.

DNA samples for probe testing were extracted from the buffy coat (BC) of the peripheral blood withdrawn from a group of 24 healthy volunteers (11 women, mean age 46.27 ± 8.47 years; 13 men, age 47.77 ± 10.03 years), employed at the University Hospital of Pisa. The volunteers were recruited during routine visits as part of a surveillance program conducted by the Occupational Medicine unit of the same hospital. All participants provided informed consent prior to inclusion in the study that was approved by the Clinical Trials Ethics Committee of Tuscany, “AREA VASTA NORD OVEST” (Protocol No. 19331, 25/02/2021).

### 2.2 Sample Processing

Peripheral blood, PFs, and STBs were collected for each patient during thoracoscopy. PFs were centrifuged at 400*g* for 15 min to separate SPF from pellet and both fractions were stored at −80°C. STB samples were directly stored at −80°C without further processing and were approximately 1 mm^3^ in volume. For ethical reasons, STBs were analyzed only in a tiny fraction available, since most of the collected material needed to be sent to the laboratory P for classical routine diagnoses.

### 2.3 BAP1 and MTAP Immunohistochemistry

BAP1 and MTAP IHC were performed on cell blocks using a mouse monoclonal anti‐BAP1 antibody (C‐4; Santa Cruz Biotechnology Inc., Dallas, TX, USA) and mouse monoclonal anti‐MTAP antibody (2G4; Abnova, Taipei City, Taiwan) both diluted 1:100, with the OptiView DAB IHC Detection Kit and OptiView Amplification Kit (Ventana Medical Systems, Tucson, AZ, USA), according to the manufacturer's protocol. Immunostaining was executed on a BenchMark XT automated slide stainer (Ventana Medical Systems). A sample was considered lacking BAP1 expression (designated “P−” for BAP1) when absence of nuclear staining was observed in all atypical mesothelial cells, in the presence of a positive internal control in inflammatory and stromal cells. Similarly, absence of cytoplasmic MTAP staining in all atypical mesothelial cells, in the presence of positive internal control, was considered indicative of MTAP loss of expression (designated “P−” for MTAP). Samples showing retained expression were classified as “P+” for BAP1 or MTAP, as appropriate.

### 2.4 p16 (*CDKN2A*) Fluorescent In Situ Hybridization (FISH)

FISH for *CDKN2A* was carried out following the manufacturers' protocols using the commercially available probe SPEC CDKN2A/CEN 9 Dual Color Probe (p16) (ZytoLight, Milan, Italy). At least 60 cells were analyzed in each case. A case was considered to show a homozygous deletion pattern when loss of *CDKN2A* signal was observed in more than 11% of atypical mesothelial cells.

### 2.5 DNA Extraction

DNA was extracted from peripheral blood and STBs from all study participants using the PureLink Genomic DNA Mini Kit (Thermo Fisher Scientific; Waltham, MA, USA). The cfDNA was extracted from SPF using the QIAamp Circulating Nucleic Acid Kit (QIAGEN, Venlo, Netherlands), according to manufacturer's instructions. DNA concentration was quantified using Qubit 3 Fluorometer (Thermo Fisher Scientific; Waltham, MA, USA).

### 2.6 Digital PCR Analysis and Test of the Probes

The test of the probes was carried out on DNA extracted from BC samples from the peripheral blood of healthy volunteers and PM patients, under the assumption that germline deletions of the investigated loci were absent in these individuals. The probes of *BAP1* (AssayID: dHsaCNS542689686, Biorad), *CDKN2A*, (AssayID: dHsaCP1000581, Biorad) *CDKN2B* (AssayID: dHsaCP1000121), and *MTAP* (AssayID: dHsaCP1000039, Biorad) were compared with the reference genes: *RPP30* (AssayID: dHsaCP2500350, Biorad), *ATRX* (AssayID: dHsaCP2506414, Biorad), *THNSL2* (AssayID: dHsaCP2506732, Biorad), *SLAIN2* (AssayID: dHsaCP2506727, Biorad) and *AP3B1* (AssayID: dHsaCP25000348, Biorad), as recommended by the Bio‐Rad protocol. DPCR reactions were performed according to the manufacturer's instructions using the QIAcuity Probe PCR Kit (Qiagen, Germany) and were run on a Qiacuity One instrument (Qiagen, Germany). A probe was considered performing accurately when, tested on control samples, its ratio with the reference gene provided a distribution of values whose average, ±1 standard deviation (SD), that included the expected diploid value of 2.

**Figure 1:**
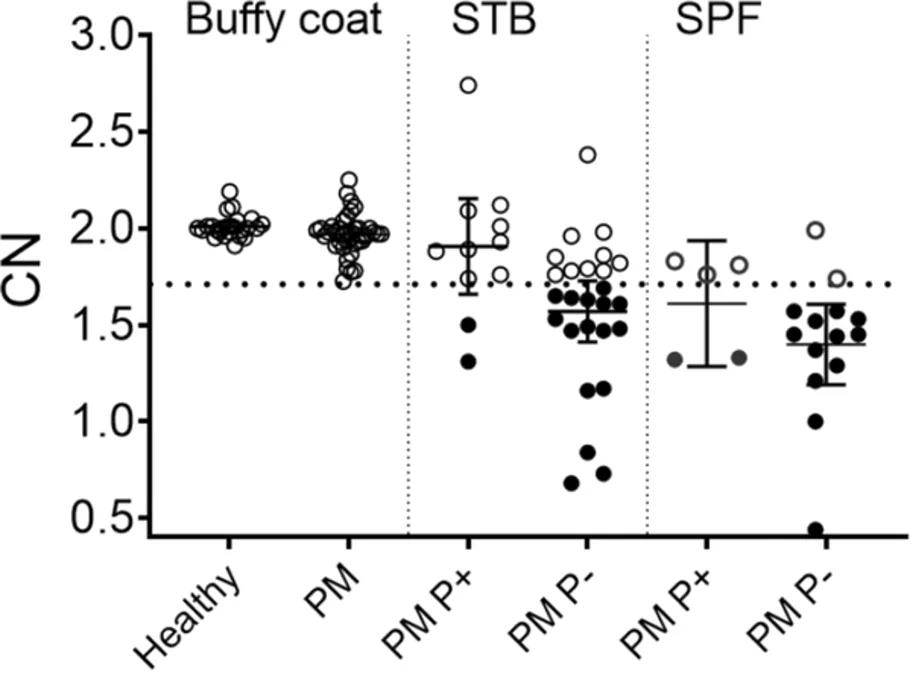
Scatterplots showing BAP1 copy number (CN) values measured by dPCR in the indicated sample types from patients with pleural mesothelioma (PM). P+ indicates retained BAP1 expression by IHC, whereas P− indicates loss of BAP1 expression, as assessed by Laboratory P. The horizontal dotted line indicates the CN threshold of 1.72 used to distinguish values within the normal diploid range from reduced CN values. Black dots indicate samples classified as having low CN.

The probe for *BAP1* was evaluated first in the control samples (BC from healthy volunteers) and the calculated CN for each subject is reported in Figure 1. This probe proved to be usable on genomic DNA, since it showed a group average of 2.01, a range of 1.91–2.19, and an SD of 0.058. BC samples from patients with PM were also evaluated and yielded similar results, with an average of 1.97, a range of 1.72–2.25, and an SD of 0.102 (Figure 1).

**Figure 2:**
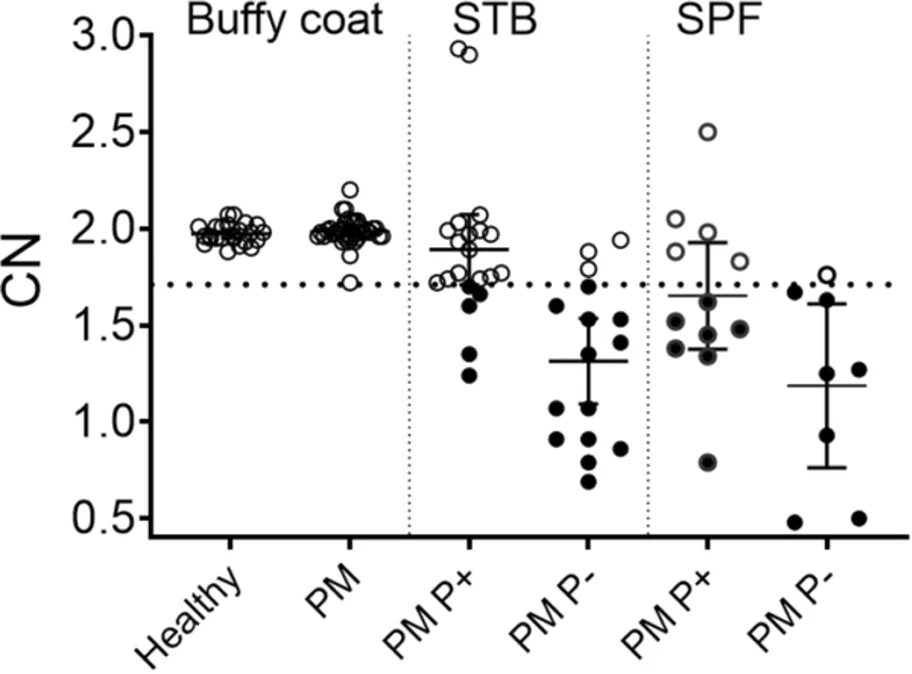
Scatterplots showing MTAP copy number (CN) values measured by dPCR in the indicated sample types from patients with pleural mesothelioma (PM). P+ indicates retained MTAP expression by IHC, whereas P− indicates loss of MTAP expression, as assessed by Laboratory P. The horizontal dotted line indicates the CN threshold of 1.72 used to distinguish values within the normal diploid range from reduced CN values. Black dots indicate samples classified as having low CN.

The *CDKN2A* probe did not show adequate performance in BC samples from healthy volunteers when using *RPP30* as a reference, because the measured CN values were systematically shifted below 2 (mean, 1.68; range, 1.24–1.95; SD, 0.212). We attempted further optimization of the assay by using other reference genes (*ATRX*, *THNSL2*, and *SLAIN2*), as suggested by previous work [38]. However, measured CN in the control samples was still offset with the different reference genes (*ATRX*: average = 1.80, range = 1.52–1.99, SD = 0.111; *THNSL2*: average = 1.84, range = 1.69–2.04, SD = 0.094; *SLAIN2*: average = 1.74, range = 1.46–1.98, SD = 0.159). Further, we tested a probe for *CDKN2B*, a gene very close to *CDKN2A* and was expected to serve as a surrogate for deletion of the *CDKN2A* locus. Here, the measured CN of *CDKN2B* was offset using *RPP30* as reference gene (average = 1.75, range = 1.46–2.05, SD = 0.145) and the offset was also observed with *AP3B1* as alternate reference gene (average =1.82, range = 1.54–1.98, SD = 0.131). On the other hand, the probe of *MTAP* showed to perform appropriately. When evaluated in the BC of healthy donors this probe showed a group average of 1.97, a range 1.88–2.07, and an SD of 0.05 (Figure 2). Similarly, using the BC from patients affected by PM, this probe showed an average of 1.98, a range of 1.72–2.20, and SD of 0.074 (Figure 2). Concerning *CDKN2A* and MTAP, FISH analysis showed 17 samples with *CDKN2A*‐loss and for 16 of them there was the correspondent lack of MTAP protein following IHC analysis. This high correspondence was also observed in several previous studies [30, 31, 36]. Therefore, we did not go further in the optimization of *CDKN2A* assay, rather, in this study, we present the results for *MTAP*, which is an established surrogate marker for *CDKN2A* homozygous deletion.

Then, in establishing a threshold for identifying samples with a reduced CN for *BAP1* and *MTAP*, we were aimed to avoid that control samples were classified as “low CN” (which corresponds to a specificity of 1 for identifying samples with normal diploidy). Therefore, we conservatively established a wide range of normality by using the lowest CN observed among all the tested BC samples. Thus, this threshold was set at 1.72, that is, at Digital PCR analysis, the CN was defined as Normal (“DN”) when ratio ≥ 1.72, and the CN was defined as Low (“DL”) when ratio < 1.72 (Figures 1 and 2). This threshold also corresponds approximately to five SDs below the expected diploid value, resulting in an extremely low theoretical probability (approximately 10^−7^) of classifying a normally diploid sample as DL.

Concordance between dPCR and IHC was assessed by classifying cases as concordant when reduced gene CN was associated with loss of protein expression (i.e., a “DLP−” status) or when normal gene CN was associated with retained protein expression (i.e., a “DNP+” status). Conversely, cases were considered discordant when reduced gene CN was associated with retained protein expression (“DLP+” status) or when normal gene CN was associated with loss of protein expression (i.e., “DNP−” status).

### 2.7 Statistical Analysis

The prevalence of low CN in PM patients as a function of the different thresholds was initially evaluated by receiver operating characteristic (ROC) curves and the evaluation of the corresponding area under the curve (AUC). Statistical analysis of the comparisons between the results obtained with dPCR and IHC was carried out with the *χ* ^2^ test. In total, 95% confidence intervals (95% CI) of the prevalence of *BAP1/MTAP* alterations among PM patients, percentage of P− samples with DL, percentage of P+ samples with DN, and of the total concordant results were calculated according to Wilson's formula. The improvement of the detection of BAP1/MTAP alterations was evaluated in 2 × 2 paired tables, and the statistical analysis was carried out with McNemar's exact test. All statistical inferences were based on two‐sided tests. Statistical analyses were carried out with Statgraphics Centurion XVI.I and with GraphPad Prism 8.

## 3 Results

### 3.1 Patient Settings, IHC, and Gene CNs

The series of analyzable patients comprised 32 men and 5 women affected by PM: 14 had ascertained exposure to asbestos, 8 were likely exposed, 2 were unexposed, while for 13 the assessment of exposure was uncertain. The mean age at diagnosis was 75.8 (±6.5 years), with a range between 60 and 87. A total of 26 patients had epithelioid PM, 10 biphasic, and 1 was sarcomatoid. Table 1 summarizes the clinic pathological findings for each of the 37 PM cases.

### 3.2 Detection of BAP1 and MTAP Alterations Using IHC on STBs, or dPCR (on STBs or SPFs), or Their Combinations

First, we evaluated the prevalence of BAP1 of MTAP alterations when assessed either by IHC alone or by dPCR alone:

a. *IHC analysis of BAP1 and MTAP in STBs*. IHC analysis of STB samples, performed by laboratory P, showed loss of BAP1 expression in 26 out of 37 patients (70.3%) and loss of MTAP expression in 16 out of 37 patients (43.2%). Cytological analysis of atypical cells detected in PFs confirmed these findings, therefore, cytological results were not further reported in this manuscript.
b. *Two‐proteins IHC signature in STBs*. The prevalence of BAP1/MTAP alterations evaluated with IHC was further assessed by establishing a simple two‐proteins signature. PM status was assigned when loss of expression of at least one of the two proteins was observed. In STB samples, IHC showed that 32 out of 37 PM patients (i.e., 86.5%; 95% CI: 72.0–94.1) presented absence of protein.
c. *dPCR analysis of BAP1 and MTAP in STBs and SPFs*. CN measurements were first evaluated by ROC curve analysis (Supporting Information S2: Figure 1). *BAP1* alone showed an AUC of 0.839 in STB samples, with a maximum Youden index of 0.694, and an AUC of 0.965 in SPF samples, with a maximum Youden index of 0.887. *MTAP* alone showed an AUC of 0.836 in STB samples, with a maximum Youden index of 0.690, and an AUC of 0.881 in SPF samples, with a maximum Youden index of 0.810. Next, we evaluated the prevalence of BAP1/MTAP alteration in PM samples, using the established threshold of 1.72. Overall, PM patients showed a high prevalence of reduced *BAP1* CN in both STB samples (18/37; 48.6%) and SPF samples (14/19; 73.7%). Reduced *MTAP* CN was also frequently observed in STB samples (18/37; 48.6%) and SPF samples (14/20; 70.0%). Gene CN data together with the corresponding IHC results, for each patient, are reported in Table 2 and shown as scatterplots in Figures 1 and 2.
d. *Two‐gene dPCR signature in STBs*. Similarly, as above, a simple two‐gene dPCR signature was established. In this case, we measured the prevalence of DL in at least one of the two genes among the patient setting. This dPCR‐STB signature showed lower sensitivity than IHC‐STB, where 27 out of 37 patients were found as DL (73.0%; 95% CI: 57.0–84.6). In the subgroup of 20 patients with available SPF samples, reduced *BAP1* and *MTAP* CNs in STBs were detected in 12/20 (60.0%) and 10/20 (50.0%) patients, respectively, whereas the two‐gene dPCR‐STB signature identified 16/20 patients (80.0%).
e. *Integrated dPCR signature in STBs and/or SPFs*. Among the 20 patients for whom both STB and SPF samples were available, a sample was considered DL when a DL state was detected in either one of the two biological samples. For the remaining 17 patients, for whom only STB was available, the state was assigned based only on STB. This approach, hereafter referred to as “integrated CN analysis,” showed a higher prevalence of DL state among PM patients (30 out of 37), corresponding to a sensitivity of 81.1% (95% CI: 65.8–90.5). This improvement was mainly attributable to the high performance in the subgroup of 20 patients with available SPF samples, in which dPCR‐SPF alone could show a DL state for 18 out of 20 patients (90.0%; 95% CI: 69.9–97.2). In the same subgroup, IHC‐STB showed loss of BAP1/MTAP protein for 17 of them (85.0%; 95% CI: 64.0–94.8) (Table 3). Overall, the comparison between IHC‐STB two‐protein signature and the integrated CN analysis showed no statistically significant difference, suggesting similar prevalence of protein or genetic alterations.

**Table 2:** Results obtained laboratory P aside from those obtained with dPCR.

	BAP1 (P)	MTAP (P)	*BAP1* (*dPCR*)		*MTAP* (*dPCR*)	
	STB	STB	STB	SPF	STB	SPF
1	−	−	L	L	N	L
2	−	−	L	L	L	L
3	−	−	L		L	
4	−	+	L		N	
5	−	+	N	L	N	N
6	+	+	N		N	
7	−	−	L	N/A	N	L
8	+	+	N	N	N	L
9	−	+	N		N	
10	−	+	N	L	L	L
11	+	−	L	L	L	L
12	+	+	N	L	L	L
13	+	−	N		L	
14	−	+	N		N	
15	+	−	N		L	
16	+	−	N		L	
17	−	+	N		N	
18	−	+	N		N	
19	−	+	L	N	N	N
20	+	+	L		N	
21	+	−	N		L	
22	−	+	L	L	N	N
23	+	−	N	N	L	L
24	−	+	L		L	
25	−	−	N		L	
26	−	−	L	L	L	L
27	−	−	N		N	
28	−	+	N	N	N	N
29	−	+	L	L	N	N
30	−	−	L	L	L	L
31	−	−	L		L	
32	−	−	L	L	L	N
33	−	+	L		N	
34	−	+	N	L	L	L
35	−	+	L	L	N	L
36	+	+	N	N	N	L
37	−	+	L	L	L	L

*Note:* “+” shows a positive nuclear staining at IHC while “*−”* shows lack of BAP1 expression according to IHC analysis. “N” are patients whose calculated CN is in the range of normal diploidy while “L” are those with a reduced CN (according to the established threshold).

**Table 3:** Prevalence of BAP1 and/or MTAP abnormalities detected by IHC and dPCR in STBs and SPFs, using individual, combined, or integrated two‐marker strategies, among 37 patients with PM, including 20 with pleural effusion.

		All PM patients (*n* = 37)		PM patients with SPFs (*n* = 20)	
Analysis	Markers	No. of correctly identified patients	Sensitivity (%)	No. of correctly identified patients	Sensitivity (%)
IHC	BAP1 (−)	26	70.3 (54.2–82.5)	15	75 (53.1–88.8)
IHC	MTAP (−)	16	43.2 (28.7–59.1)	8	40 (21.9–61.3)
IHC	BAP1 + MTAP	32	86.5 (72–94.1)	17	85 (64–94.8)
dPCR in STB	*BAP1* (DL)	18	48.6 (33.4–64.1)	12^a^	60 (38.7–78.1)
dPCR in STB	*MTAP* (DL)	18	48.6 (33.4–64.1)	10	50 (29.9–70.1)
dPCR in STB	*BAP1* + *MTAP*	27	73.0 (57–84.6)	16	80 (58.4–91.9)
dPCR in SPF	*BAP1* (DL)			14^a^	73.7 (51.2–88.2)
dPCR in SPF	*MTAP* (DL)			14	70 (48.1–85.5)
dPCR in SPF	*BAP1* + *MTAP*			18	90 (69.9–97.2)
dPCR integrated STB + SPF	*BAP1* + *MTAP*	30	81.1 (65.8–90.5)		
IHC + dPCR in STB	BAP1 + MTAP	34	91.9 (78.7–97.2)	18	90 (69.9–97.2)
IHC + dPCR in STB & SPF	BAP1 + MTAP			20	100 (83.9–100)
IHC + dPCR in SPF	BAP1 + MTAP			20	100 (83.9–100)
IHC + dPCR integrated STB + SPF	BAP1 + MTAP	36	97.3 (86.2–99.5)		

a One sample failed the analysis for BAP1 (the total is *n* = 19).

Then, the two proteins IHC‐STB signature was evaluated in combination with two‐gene CN signature, according to the following schemes:

a. *IHC‐STB signature + dPCR‐STB*. In STB samples, the capacity to detect BAP1/MTAP alterations (defined as either loss of protein expression or DL for *BAP1* or *MTAP*) increased from 32 to 34 (91.9%; 95% CI: 78.7–97.2) when the IHC signature was combined with that of CN analysis.
b. *IHC‐STB signature + dPCR‐SPF*. Among 20 PM patients presenting PFs, the prevalence of BAP1/MTAP abnormalities defined by IHC‐STB signature could be increased by adding the dPCR‐SPF signature, allowing to detect at least one alteration in all the patients (from 17/20 to 20/20).
c. *IHC‐STB signature *+* integrated CN analysis*. When IHC‐STB signature was combined with the integrated CN analysis, almost all PM patients (36 out of 37, 97.3%) presented at least one BAP1/MTAP alteration.

### 3.3 Comparison Between *BAP1* and *MTAP* dPCR and IHC

*BAP1 abnormalities:* For *BAP1*, the comparison between dPCR and IHC on STB samples showed concordant results in 25 out of 37 patients (67.6%). Specifically, 16 patients were classified as DLP−, whereas 9 were classified as DNP+. Discordant results were observed in 12 out of 37 patients, including 2 DLP+ and 10 DNP− cases. The association between dPCR and IHC results was statistically significant (*p* = 0.0159).

When dPCR‐SPF was compared with IHC‐STB, concordant results were observed in 15 out of 19 patients (78.9%), including 12 DLP− and 3 DNP+ cases. Four patients (21.1%) showed discordant results, consisting of 2 DNP− and 2 DLP+ cases.

*MTAP abnormalities:* For *MTAP*, the comparison between dPCR and IHC on STB samples showed concordant results in 29 out of 37 patients (78.4%). Specifically, 13 patients were classified as DLP−, whereas 16 were classified as DNP+. Discordant results were observed in 8 out of 37 patients, including 5 DLP+ and 3 DNP− cases. The association between dPCR and IHC results was statistically significant (*p* = 5 × 10^−4^).

When SPF‐dPCR was compared with IHC‐STB, concordant results were observed in 12 out of 20 patients (60.0%), including 7 DLP− and 5 DNP+ cases. Eight patients (40.0%) showed discordant results, consisting of 1 DNP− and 7 DLP+ cases. The results are reported in Table 4.

**Table 4:** Comparison of BAP1 and MTAP abnormalities detected by IHC or dPCR.

	dPCR, STB		dPCR, SPF		dPCR, STB		dPCR, SPF	
	DL	DN	DL	DN	DL	DN	DL	DN
P, STB								
P−	16	10	12	2	13	3	7	1
P +	2	9	2	3	5	16	7	5
*N*	37		19		37		20	
*p*	0.0159		0.0463		5 × 10‐4		0.163	
%P− with DL	61.5	42.5–77.6	85.7	60.1–96.0	81.3	57.0–93.4	87.5	52.9–97.8
%P+ with DN	81.8	52.3–94.9	60	23.1–88.2	76.2	54.9–89.4	41.7	19.3–68.0
Concord. (%)	67.6	51.5–80.4	78.9	56.7–91.5	78.4	62.8–88.6	60	38.7–78.1

*Note:* STBs were analyzed by Laboratory P using IHC, and the results were compared with those obtained by dPCR using DNA extracted from STBs or the corresponding SPFs. P+ indicates retained protein expression, whereas P− indicates loss of protein expression by IHC. DL indicates a gene copy number below the established threshold relative to the diploid reference gene *RPP30*, whereas DN indicates a copy number within the normal diploid range. The reported percentages indicate the proportion of P− samples classified as DL and the proportion of P+ samples classified as DN. Concordance was calculated as the proportion of samples classified as either DLP− or DNP+.

### 3.4 Proof of Concept on a Small Prospective Series of High‐Risk People for PM

We applied the two‐genes signature to a small series of 6 people presenting at thoracoscopy because of a PE and suspected for PM given their past exposure to asbestos. The laboratory P was unable to detect any malignant disease thus, at the time of the thoracoscopy, these people were found negative for PM using the classical IHC biomarkers, thus considered affected by an unspecified phlogosis and the IHC analysis of BAP1 and MTAP on STBs was not carried out. However, these individuals underwent clinical follow‐up, and their main outcomes are reported in Table 5 (additional details on clinical follow‐up are provided in Supporting Information S1: Table 1). For two of them, a novel diagnosis of PM was delivered after 6‐7 months from the initial thoracoscopy. Interestingly, in both patients, reduced CN involving at least one of the two genes was already detectable in the SPF collected at that time.

**Table 5:** Main information about patients affected by an unspecified inflammatory state.

Code ID	Date of thoracoscopy (age)	Duration of the follow‐up (months)	Diagnosis at follow‐up	*BAP1*	*MTAP*
001	12/12/2020 (74)	11	Healthy	N	N
004	30/01/2023 (83)	15	Chronic inflammation	N	N
008	17/07/2023 (76)	8	Healthy	N	N
009	03/08/2023 (79)	26	Healthy	N	N
012	09/07/2024 (70)	7	PM	L	L
015	13/12/2024 (76)	6	PM	N	L

## 4 Discussion

The main goal of the present study was to determine the prevalence of low *BAP1* and *MTAP* CNs in STBs and cfDNA extracted from SPFs of patients with PM. dPCR results obtained in STBs were compared with the corresponding BAP1 and MTAP immunohistochemical findings. We also evaluated the detection yield of strategies based on IHC alone, dPCR alone, or combined and integrated analyses of the two methods. In a cohort of 37 patients with PM, the two‐protein IHC signature detected loss of BAP1 and/or MTAP expression in 86.5% of cases. Cytological analyses of PF cells were largely concordant with the corresponding STB findings and did not appear to increase detection beyond that achieved by IHC in STBs. Previous studies of pleural [27] and peritoneal mesothelioma [36] effusions reported slightly lower sensitivities for cytological assessment. *BAP1* and *MTAP* CNs were then measured in STBs and SPFs and assessed as a two‐gene signature and as part of an integrated dPCR‐plus‐IHC strategy. Gene‐CN analysis yielded high AUC values in ROC analyses, and the prevalence of abnormalities detected by the two‐gene CN signature was comparable to that observed with the two‐protein IHC signature in STBs. Combining IHC and CN analysis increased the proportion of patients with at least one detected abnormality. Among the 20 patients with PE for whom both analyses were available, combining dPCR analysis of SPFs with IHC analysis of STBs increased detection from 17/20 to 20/20. In the full cohort, the proportion increased from 86.5% to 97.3% (36/37). dPCR analysis of SPF‐derived cfDNA alone detected low CN involving at least one of the two genes in 18/20 patients. We considered whether this high detection rate could reflect specimen‐specific matrix effects or differences in disease severity between patients with and without PE. Probe testing and threshold setting were performed using genomic DNA from BC samples, whereas the assay was subsequently applied to cfDNA. In addition, SPF samples from individuals without cancer were not systematically available, precluding the establishment of a diploid reference range in the same specimen type. SPF‐derived DNA may differ from BC DNA in fragmentation or in the presence of matrix‐associated substances that affect probe performance. However, the few available noncancer SPF samples showed no systematic reduction in *BAP1* or *MTAP* CN, and normal values were also observed in some SPF samples from patients with PM. These findings argue against a major systematic matrix effect, although larger series of noncancer PEs are required to define specimen‐specific reference values. The high rate of CN abnormalities in SPFs was also unlikely to be explained solely by greater disease severity, because patients with PE showed a similar or slightly lower prevalence of IHC abnormalities in STBs than patients without effusion. Thus, in this cohort, dPCR analysis of SPFs yielded a detection rate similar to or higher than that obtained by IHC in STBs.

Concordance between IHC and dPCR in STBs was 67.6% for BAP1 and 78.4% for MTAP. These findings are broadly consistent with a previous study comparing *BAP1* and *CDKN2A* DNA losses detected by dPCR with BAP1 IHC and *CDKN2A* FISH, respectively [36], and support the feasibility of dPCR‐based CN assessment in PM tissue samples. The discordant DNP− and DLP+ profiles also indicate that CN analysis and IHC provide partly complementary information. DNP− cases may result from point mutations, small insertions or deletions, epigenetic alterations, or other events that impair protein expression without reducing gene CN. DLP+ cases may reflect sampling variability, intratumoral heterogeneity, or CN loss in tissue areas represented in the specimen analyzed by dPCR but not in the section evaluated by IHC. DNA was not extracted from the same tissue sections used for IHC, and tumor‐enriched microdissection was not performed. Consequently, differences in tumor‐cell content and spatial representation between the samples used for the two assays may have contributed to the observed discordance and should be addressed in future studies through matched‐section analysis or tumor‐enriched sampling.

A modest reduction in *BAP1* or *MTAP* CN in SPF‐derived cfDNA may therefore reflect a homozygous deletion in a subpopulation of neoplastic cells diluted by DNA from non‐neoplastic cells. Alternatively, it may reflect a hemizygous deletion in which the remaining allele still produces a protein detectable by IHC. Conversely, retained staining does not necessarily establish preserved wild‐type function. Reduced CN alone should therefore not be regarded as direct evidence of malignancy or as a definitive biomarker of PM.

Nevertheless, reduced CN may indicate a clonal genetic alteration involving a tumor‐suppressor locus. According to Knudson's two‐hit model, hemizygous loss may represent an early event preceding complete functional inactivation. Reduced CN in a pleural effusion may also originate from metastatic involvement by another primary tumor. CN measurements should therefore be interpreted together with epidemiological features, cytomorphology, immunocytochemical markers, and established molecular analyses within an integrated weight‐of‐evidence framework. In patients at increased risk of PM, including those with previous asbestos exposure, suspicious imaging findings, or recurrent pleural effusion, CN analysis might identify abnormalities warranting further investigation and closer follow‐up. Timely diagnosis remains challenging, and patients with pleural effusion and nonspecific pleuritis may subsequently receive a diagnosis of PM within 32 months [39, 40, 41]. The possible role of CN analysis in risk stratification therefore requires validation in larger prospective studies with appropriate noncancer controls and longitudinal follow‐up.

As preliminary evidence, we prospectively evaluated six patients considered at risk of PM because of previous asbestos exposure and abnormal chest imaging. All had pleural effusion but no evidence of PM at initial thoracoscopy. SPF samples from two patients showed reduced CN involving *BAP1* and/or *MTAP*, and both received a diagnosis of PM approximately 6 months later. This observation is consistent with previous studies in which BAP1‐ and *CDKN2A*‐related abnormalities were detected in cytological specimens several months before a definitive diagnosis [42, 43]. Although this very small series is only hypothesis‐generating and does not establish predictive performance, it supports further investigation of SPF analysis in patients with an initially negative thoracoscopic assessment but persistent clinical or radiological suspicion.

Several methodological developments could improve sensitivity. The threshold used to define normal CN was deliberately conservative and was established using BC DNA from both healthy volunteers and patients with PM. Because PM patients showed slightly lower CN values than healthy volunteers, their inclusion in the reference group reduced the likelihood of false‐positive classifications but may have favored specificity at the expense of sensitivity. In addition, only two genes were analyzed. Expanding the panel to include other loci frequently affected by copy‐number loss in PM, such as *NF2* [27, 44], could increase the detection yield. Other limitations include the small sample size, limited statistical power, and the inability to perform histotype‐specific analyses. Overall, these findings provide preliminary proof‐of‐concept evidence that dPCR‐based CN analysis of DNA extracted from PF may complement tissue‐based IHC and support further diagnostic assessment in selected high‐risk or unresolved cases. Validation in larger, prospectively characterized cohorts, including patients with benign and metastatic pleural disease, is required to establish specimen‐specific reference ranges, diagnostic specificity, predictive value, and the most appropriate clinical setting before implementation.

## Author Contributions

Conception and design of the study: Stefano Landi, Federica Gemignani, Marco Lucchi, Greta Alì, and Rudy Foddis. Acquisition and analysis of data: Giulia Rosini, Vittorio Aprile, Rossella Bruno, Alessandra Celi, Filomena Rea, and Monica Cipollini. Drafting the manuscript or figures: Giulia Rosini, Monica Cipollini, Roberto Silvestri, Stefano Landi, and Federica Gemignani.

## Ethics Statement

This research was approved by the Clinical Trials Ethics Committee of Tuscany, “AREA VASTA NORD OVEST” (Protocol No. 19331, 25/02/2021).

## Consent

All participants provided informed consent prior to inclusion in the study.

## Conflicts of Interest

The authors declare no conflicts of interest.

## Supporting information


Supporting File 1

Supporting File 2

## Data Availability

The data that support the findings of this study are available from the corresponding author upon reasonable request.
