# Supplementary material for: Digital PCR for Detecting Loss of BAP1 and MTAP in Pleural Fluids of Mesothelioma Patients: Perspectives in the Diagnosis
Source: Pathol Int. 2026 Sep 27;76(9):e70172. doi: 10.1111/pin.70172 (PMC13616427; doi:10.1111/pin.70172)
Supplement: Supplementary file 1 [file PIN-76-0-s002.docx]

**Supplementary Table 1.** Details on clinical follow-up of patients affected by an unspecified inflammatory state.

| **Case** | **Thoracoscopic Histology** | **Cytology** | **Immunohistochemistry (IHC)** | **Imaging Findings** | **Follow-up Interval** | **Trigger for Re-evaluation** |
| --- | --- | --- | --- | --- | --- | --- |
| **001** | DATA: 15/12/2020  Pleural tissue fragments with marked fibrosis and minimal chronic lymphomonocytic inflammation. | Blood and sparse inflammatory cells. | Not performed. | PET/CT demonstrated focal pleural uptake. | Last follow-up at 3 months after surgery showed no recurrent pleural effusion and effective pleurodesis. | Chest pain |
| **004** | DATA: 30/01/2023  Pleural tissue fragments showing diffuse chronic lymphoplasmacytic inflammatory infiltrates with histiocytic aggregates, associated with mild granulation tissue formation and fibrosclerotic changes. | Inflammatory cells with occasional reactive mesothelial cells. | Not performed. | CT and PET imaging demonstrated a hypermetabolic pleural lesion infiltrating the chest wall. | CT at 3 months and PET at 5 months after surgery. | Chest pain |
| **008** | DATA: 17/07/2023  Pleural tissue fragments exhibiting chronic lymphoplasmacytic inflammation associated with fibrotic changes and reactive mesothelial hyperplasia. | Occasional inflammatory cells. | Not performed. | Chest radiography at 6 months demonstrated persistent pleural effusion. | Radiologic follow-up at 6 months. | Dyspnea |
| **009** | DATA: 3/8/2023  Pleural tissue fragments showing focal fibrin deposition, mixed acute and chronic inflammatory infiltrates, and exuberant granulation tissue with fibroblastic/fibrotic organization. Focal reactive mesothelial hyperplasia with mild cytonuclear atypia. Adjacent lung parenchyma showed focal organizing pneumonia. | Blood, inflammatory cells, and histiocytes. | Not performed. | CT at 6 months demonstrated regular postoperative findings without pleural effusion and effective pleurodesis. | Monthly chest radiographs until CT evaluation at 6 months. | None |
| **012** | DATA: 10/07/2024  Initial biopsy: fibrin deposition, fibrotic changes, and mild focal reactive mesothelial hyperplasia.  DATA: 13/02/2025  Repeat biopsy: focal proliferation of epithelioid mesothelial cells with moderate atypia within fibrous stroma, suggestive of low-grade epithelioid mesothelioma. | Blood, fibrinoid material, and inflammatory cells. | 10/07/2024: no evidence of PM  13/02/2025: Positive for pan-CK, CK5/6, calretinin, WT1, and EMA; negative for podoplanin, BerEP4, TTF-1, and CEA. Loss of nuclear BAP1 expression. Ki-67 ≈60%. PD-L1 TPS 50%. | 13/02/2025: CT at 6 months demonstrated bilateral pleural and pulmonary lesions following investigations for dyspnea and trapped lung. | From 10/07/2024, monthly chest radiography followed by CT scan. After ~6 months: PET imaging, and repeat surgery. | Dyspnea |
| **015** | DATA: 16/12/2024  Fragments of pleural tissue with diffuse fibro-sclerosis, foci of granulation tissue, and inflammatory infiltrates predominantly composed of lymphomonocytic and plasma cells, occasionally with follicular organization. Within the thickness of the fibrous tissue, occasional nests of epithelioid cells are identified, showing mild cytonuclear atypia, most likely consistent with reactive mesothelial cells entrapped as a consequence of previous inflammatory processes. | Inflammatory cells only. | Immunohistochemical studies: CK-pan, calretinin, WT1, podoplanin, and BAP1 positive in reactive mesothelial cells; BerEP4, TTF1, and CEA negative. | Chest radiography at 6 months: demonstrated pleural effusion and pulmonary consolidations with mediastinal shift. Worsening of health conditions preventing novel thoracoscopy. Pleural involvements compatible with pleural mesothelioma. | Monthly Chest X-Ray | Dyspnea |
